# A clinical–radiomics nomogram based on multisequence MRI for predicting the outcome of patients with advanced nasopharyngeal carcinoma receiving chemoradiotherapy

**DOI:** 10.3389/fonc.2024.1460426

**Published:** 2024-11-20

**Authors:** Liucheng Chen, Zhiyuan Wang, Ying Meng, Cancan Zhao, Xuelian Wang, Yan Zhang, Muye Zhou

**Affiliations:** ^1^ Department of Radiology, The First Affiliated Hospital, Bengbu Medical University, Bengbu, Anhui, China; ^2^ School of Medical Imaging, Bengbu Medical University, Bengbu, Anhui, China

**Keywords:** nasopharyngeal carcinoma, magnetic resonance imaging, radiomics, nomogram, survival models

## Abstract

**Problem:**

Nasopharyngeal carcinoma (NPC) is a common malignant tumor with high heterogeneity and is mainly treated with chemoradiotherapy. It is important to predict the outcome of patients with advanced NPC after chemoradiotherapy to devise customized treatment strategies. Traditional MRI methods have limited predictive power, and better predictive models are needed.

**Aim:**

To evaluate the predictive value of a clinical–radiomics nomogram based on multisequence MRI in predicting the outcome of advanced NPC patients receiving chemoradiotherapy.

**Methods:**

This prospective study included a retrospective analysis of 118 patients with advanced NPC who underwent MRI prior to chemoradiotherapy. The primary endpoint was progression-free survival (PFS). The maximum ROIs of lesions at the same level were determined via axial T2-weighted imaging short-time inversion recovery (T2WI-STIR), contrast-enhanced T1-weighted imaging (CE-T1WI), and diffusion-weighted imaging (DWI) with solid tumor components, and the radiomic features were extracted. After feature selection, the radiomics score was calculated, and a nomogram was constructed combining the radiomics score with the clinical features. The diagnostic efficacy of the model was evaluated by the area under the receiver operating characteristic curve (AUC), and the clinical application value of the nomogram was evaluated by decision curve analysis (DCA) and a correction curve. Patients were divided into a high-risk group and a low-risk group, and the median risk score calculated by the joint prediction model was used as the cutoff value. Kaplan−Meier analysis and the log-rank test were used to compare the differences in survival curves between the two groups.

**Results:**

The AUCs of the nomogram model constructed by the combination of the radiomics score and neutrophil-to-lymphocyte ratio (NLR) and T stage in the training group and validation group were 0.897 (95% CI: 0.825–0.968) and 0.801 (95% CI: 0.673–0.929), respectively. Kaplan-Meier survival analysis demonstrated that the model effectively stratified patients into high- and low-risk groups, with significant differences in prognosis.

**Conclusion:**

This clinical–radiomics nomogram based on multisequence MRI offers a noninvasive, effective tool for predicting the outcome of advanced NPC patients receiving chemoradiotherapy, promoting individualized treatment approaches.

## Introduction

Nasopharyngeal carcinoma (NPC) is one of the most common malignant tumors of the head and neck. In 2020, approximately 133,000 new cases and 80,000 deaths were reported worldwide, and the incidence and mortality of NPC in China were significantly higher than the global average (1–3). NPC is a highly heterogeneous malignant tumor, and the choice of treatment is mainly based on the tumor node metastasis (TNM) staging system (4). The current standard treatment strategy is concurrent chemoradiotherapy (CCRT) combined with or without adjuvant chemotherapy (AC) and induction chemotherapy (IC) (5). However, this staging system has many shortcomings in predicting the treatment effect and outcome of patients (6, 7). Nor does it accurately reflect the likelihood of metastasis and continued invasiveness after treatment. Especially for patients with advanced NPC, even patients with the same TNM stage have different progression-free survival (PFS) rates after receiving chemoradiotherapy (8).

In recent years, several biomarkers, such as the plasma protein (9), neutrophil-to-lymphocyte ratio (NLR) (10), gene expression marker (11), and EB virus (12) levels, have been shown to be highly important for predicting the outcome of patients with NPC. However, these indicators often can predict only the probability of invasion and distant metastasis and are not predictors of treatment response. Therefore, a better method to predict the PFS of NPC patients after receiving chemoradiotherapy is urgently needed to allow for the formulation of an optimal treatment strategy and to adjust the individualized treatment regimen in a timely manner. The rise of radiomics, which can extract high-dimensional quantitative features that cannot be recognized by the naked eye from images, provides a new approach to overcoming this challenge (13).

The emergence and development of radiomics technology provides the possibility of realizing individualized diagnosis and treatment of tumors. Among them, in the field of NPC research, many studies have shown that radiomic features based on MRI are highly important for evaluating treatment efficacy and predicting patient outcome (14–18). Zhang et al. (17) accurately predicted the outcome of patients with advanced NPC through a multiparameter MRI radiomics nomogram, providing a useful example for precision medicine. Kim et al. (18) established a survival model based on MRI radiomics to conduct early risk assessments by predicting the PFS of NPC patients. The accuracy of the clinical + stage + radiomics survival model was better than that of the clinical TNM stage. Therefore, we aimed to establish a nomogram model for predicting the PFS of patients with advanced NPC after chemoradiotherapy by combining multisequence MRI with clinical risk factors to optimize individual treatment strategies for better survival outcomes.

The contribution of our study is threefold. First, we selected a high-throughput method to extract radiomic features from the MR images of patients with advanced NPC and combined these with clinical risk factors to establish a prediction model based on a multisequence MRI clinical−radiomics nomogram, which can predict the outcome of patients with advanced NPC after chemoradiotherapy earlier. Second, compared with other radiomic studies in the field of NPC, we added DWI sequences and extracted important radiomic features from them, which is highly important for prognostic models. Finally, we combined T stage, the NLR and the radiomic features to develop a more comprehensive prognostic model.

The rest of this paper is organized as follows: In the second part, we introduce the general information, examination methods and specific treatment strategies, as well as the specific steps to implement this study. In the third part, we obtain the results of this study and analyze the results. Finally, we discuss the methods and results of this study and explain its practical significance and shortcomings.

## Methods

### Study population

**Figure 1:**
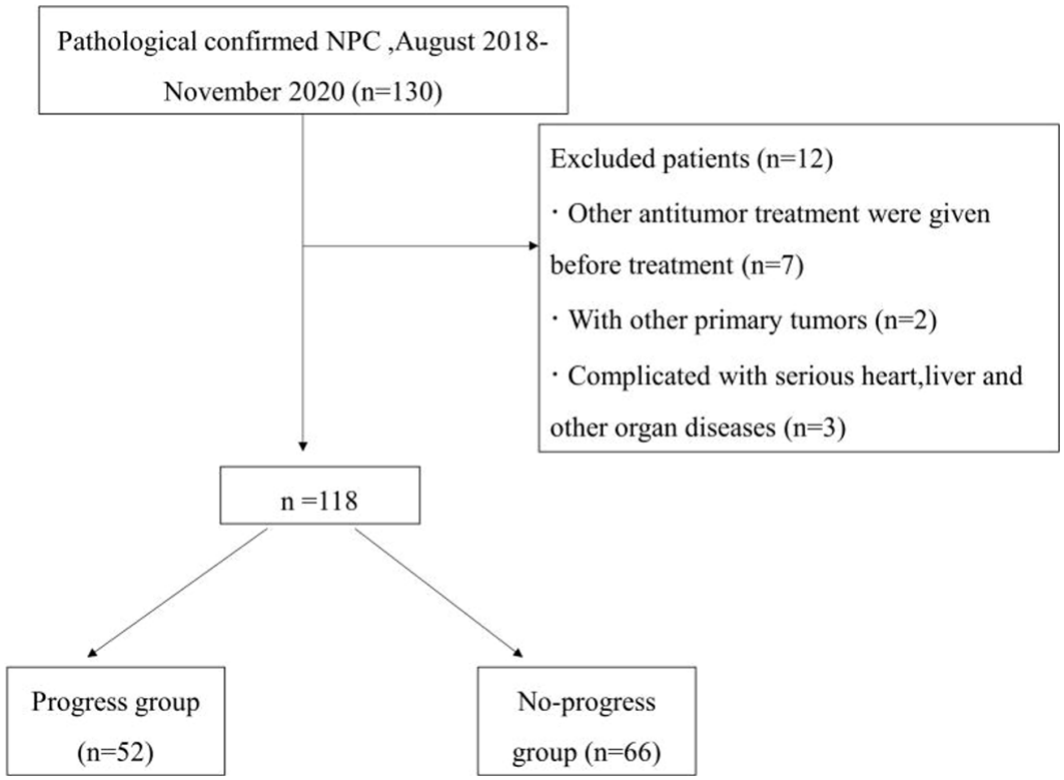
Flowchart of patient selection.

A retrospective analysis was performed on 118 patients newly diagnosed with advanced NPC on the basis of pathology at the First Affiliated Hospital of Bengbu Medical University from August 2018 to November 2020. The detailed patient inclusion criteria are illustrated in **Figure 1**. The First Affiliated Hospital of Bengbu Medical University Ethics Committee approved this study, which was conducted according to all relevant regulations. This retrospective study was exempt from the need for informed consent (Ethical Approval Number: 2023YJS158).

The inclusion criteria were as follows: (1) clinical stage II–IV (AJCC 8th edition); (2) received a 3.0 T magnetic resonance examination within 2 weeks before treatment; (3) intensity-modulated radiotherapy (IMRT) with the following chemotherapy modalities: CCRT, IC+CCRT, AC+CCRT; and (4) complete follow-up data.

The exclusion criteria were as follows: (1) received other antitumor therapy before treatment; (2) had other primary tumors; and (3) had severe heart, liver or other organ diseases.

### Treatment and follow-up

Intensity-modulated radiation therapy (IMRT) was used: Each patient received an IMRT plan according to the Chinese Guidelines for Radiotherapy for NPC (2020 edition) or our hospital standards [nasopharyngeal primary site prescription dose: planning target volume (PTV)-gross tumor volume (GTV) |nx|, total dose (DT) 68−76 Gy/30−33 cycles; PTV-clinical target volume 1 (CTV1), DT60−64 Gy/30−33 cycles; PTV-CTV2, DT50−54 Gy/30−33 cycles]. For concurrent chemotherapy, patients received cisplatin monotherapy starting from Day 1 of radiotherapy with an intravenous drip at a dose of 100 mg/m2 and ending at the same time as radiotherapy. For patients with advanced NPC (stage III~IV), CCRT was combined with IC or AC. The IC consisted of 2 cycles and was repeated every 21 days. The chemotherapy regimen was based on platinum (cisplatin/nedaplatin), and the common IC regimens used include docetaxel + cisplatin + fluorouracil (TPF), gemcitabine + cisplatin (GP), and cisplatin + fluorouracil (PF), among others. The ACs were subjected to TPF or PF regimens once every 21 days for a total of 1~3 cycles.

PFS was defined as the period from randomization to disease progression, death, or cutoff date (November 30, 2023), with a minimum follow-up of 36 months. Patients were divided into a progression group (disease progression, death) and a nonprogression group on the basis of the follow-up results. The patients were randomly divided into a training group (n=70, of which 31 were in the progression group and 39 were in the nonprogression group) and a validation group (n=48, of which 21 were in the progression group and 27 were in the nonprogression group) at a ratio of 6:4.

### MRI image acquisition

A Philips Achieva 3.0 T double gradient superconducting magnetic resonance instrument (The Netherlands) was used. Gadobenate meglumine was used as the contrast agent for enhanced scanning. Image postprocessing was performed via ITK-SNAP and AK software. A 16-channel combined head and neck coil was used to scan from the skull base to the thoracic entrance. The scan sequences included T1-weighted imaging (T1WI), T2WI-short-time inversion recovery (T2WI-STIR), DWI, sagittal T1WI, and coronal, sagittal, and axial contrast-enhanced T1-weighted imaging (CE-T1WI) scan sequences. For DWI, the excitation time was 1, the b value was 0 s/mm² or 1000 s/mm². The contrast agent was subsequently injected into the cubital vein at a total amount of 0.1 mmol/kg at a rate of 2 ml/s, and a multidirectional T1WI-STIR enhanced examination was subsequently performed. The detailed scanning parameters of each sequence are shown in **Table 1**.

**Table 1:** Scanning parameters for various scan sequences.

Scan Sequence	TR (ms)	TE (ms)	FOV	Layer Thickness	Layer Spacing	Layer Number
T_2_WI-STIR	7620	60	230*260	2 mm	0.5 mm	37
CE-T_1_WI	500	8	240*240	2 mm	0.5 mm	37
DWI	5200	70	20*20	2 mm	0.5 mm	–

### Radiomic features extraction

**Figure 2:**
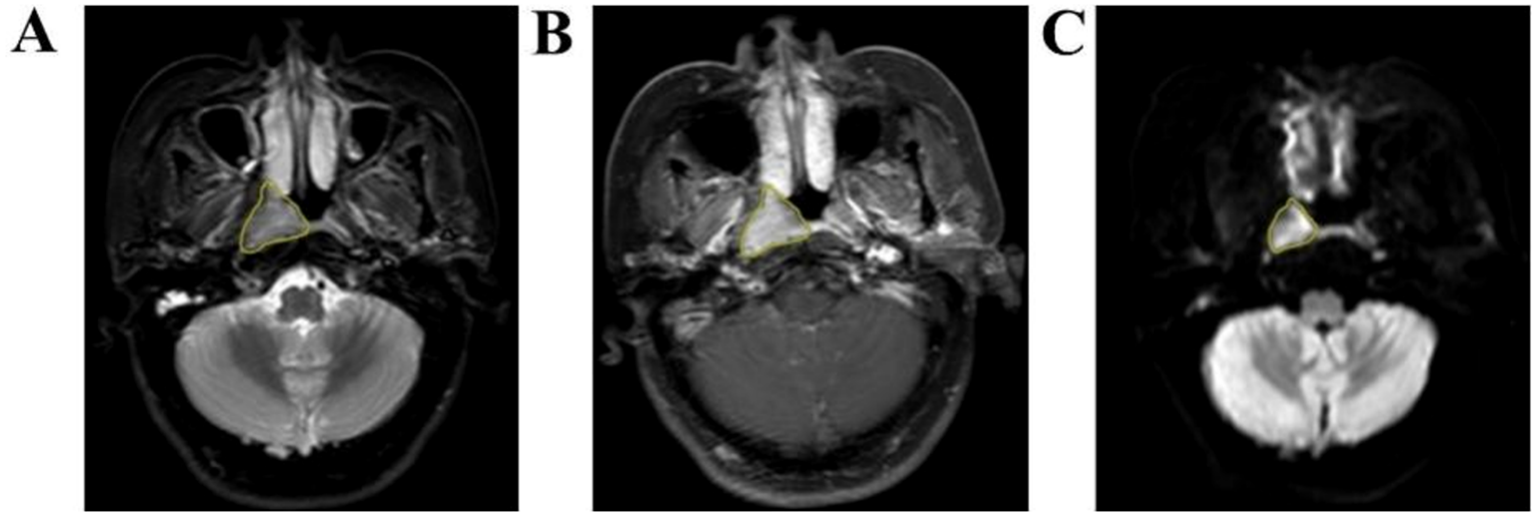
Manual outline of the ROI schematic at the maximum tumor level. **(A–C)**: Results of manual lesion segmentation on T2WI-STIR, CE-T1WI and DWI images, left to right.

Axial T2WI-STIR, CE-T1WI and DWI images of patients were imported into the medical-Darwin platform in the original image format (DICOM), and manual segmentation was adopted. In the software, the region of interest (ROI) was delineated for the largest lesions at the same level, showing solid tumor components on axial T2WI-STIR, CE-T1WI and DWI (**Figure 2**). After all of the lesions were sketched, the ROI of the sketched area of interest was imported into the relevant platform to extract the radiomic features, such as exponential, logarithm, square, square root, gradient, local binary pattern-2-dimensional (LBP-2D), and log-sigma features, which were extracted from the original multimodal MR images to create transformed images. The second-order features included a gray-level concurrence matrix (GLCM), gray-level size zone matrix (GLSZM), gray-level run length matrix (GLRLM), neighborhood gray tone difference matrix (NGTDM), and gray-level dependency matrix (GLDM).

### Feature selection

The minimum maximum normalization method and Select K Best were used to select the features and remove the low-performance, redundant, and irrelevant features. Finally, least absolute shrinkage and selection operator (LASSO) was used to further reduce the dimension and screen the best features with nonzero coefficients. The eigenvalues and regression equation coefficients were linearly combined to obtain a Rad score for each patient.

### Model construction

In the training group, clinical risk factors such as age, tumor length, sex, the NLR and the platelet-to-lymphocyte ratio (PLR) of each patient were analyzed via backward stepwise logistic regression, and a clinical information model was constructed. The radiomics score and clinical information were then combined, and RStudio software was subsequently used to construct a nomogram to visualize the prediction model. The ROC curve was used to evaluate the ability of the joint prediction model to differentiate between the training set and the validation set, and the AUC was compared with that of the clinical information model and the radiomics model. The calibration curves of the training set and validation set were drawn via the bootstrap resampling method to evaluate the degree of calibration of the prediction model and compare the consistency of the model-predicted probability and observed probability. Decision curve analysis (DCA) was performed on the prediction model in the training set and validation set to evaluate the clinical practicability of the model and to compare it with the clinical indicator model and the radiomics model.

### Statistical analysis

SPSS 26.0 and RStudio software were used for the statistical analyses. The Kolmogorov−Smirnov test was used to test the normality of the quantitative data (age and tumor length and diameter). The normally distributed data are presented as the means ± standard deviations, and the independent sample t test was used for comparisons between two groups. Qualitative data (sex, presence of nodules, presence of lymph node metastasis at first diagnosis, etc.) are presented as n (%). The chi-square test or Fisher’s exact probability method was used to compare the two groups. The prediction model constructed via Cox proportional risk regression was used to calculate the risk score of each patient, and the patients were divided into a high-risk group and a low-risk group, with the median risk score as the cutoff value. The Kaplan−Meier curve and log-rank test were subsequently used to analyze the survival of the high-risk group and low-risk group. P < 0.05 was considered to indicate statistical significance.

## Results

### Patient clinical information

No statistically significant differences in the PLR, age, tumor length, sex, presence or absence of bloody nasal discharge, presence or absence of lymph node metastasis at first diagnosis, or clinical stage were observed between the training group and the validation group (P > 0.05, **Table 2**).

**Table 2:** Clinical information of the patients.

Index	Training set			Validation set		
	Nonprogression group	Progression group	P value	Nonprogression group	Progression group	P value
n	39	31		27	21	
NLR (*Mean ± SD*)	2.522 ± 1.038	3.131 ± 1.319	0.034	2.910 ± 0.844	3.469 ± 1.457	0.102
PLR (*Mean ± SD*)	172.730 ± 107.879	217.685 ± 185.216	0.208	172.936 ± 126.439	158.840 ± 83.833	0.662
Age (years, *Mean ± SD*)	47.051 ± 13.677	52.290 ± 8.661	0.068	52.519 ± 9.545	54.286 ± 13.573	0.599
Tumor length (cm, *Mean ± SD*)	3.223 ± 0.652	3.081 ± 0.520	0.325	3.207 ± 0.484	3.210 ± 0.703	0.99
Sex (%)			0.495			0.411
F	13 (33.3)	8 (25.8)		5 (18.5)	6 (28.6)	
M	26 (66.7)	23 (74.2)		22 (81.5)	15 (71.4)	
Bloody nasal discharge (%)			0.495			0.658
No	26 (66.7)	23 (74.2)		12 (44.4)	8 (38.1)	
Yes	13 (33.3)	8 (25.8)		15 (55.6)	13 (61.9)	
Lymph node metastasis (%)			0.463			0.696
No	2 (5.1)	3 (9.7)		4 (14.8)	4 (19.0)	
Yes	37 (94.9)	28 (90.3)		23 (85.2)	17 (81.0)	
T stage (%)			0.035			0.034
T1-T2	25 (64.1)	12 (38.7)		16 (59.3)	6 (28.6)	
T3-T4	14 (35.9)	19 (61.3)		11 (40.7)	15 (71.4)	
Clinical stage (%)			0.372			0.401
II-III	29 (74.4)	20 (64.5)		19 (70.4)	17 (81.0)	
IV	10 (25.6)	11 (35.5)		8 (29.6)	4 (19.0)	

### Feature selection and model building

**Figure 3:**
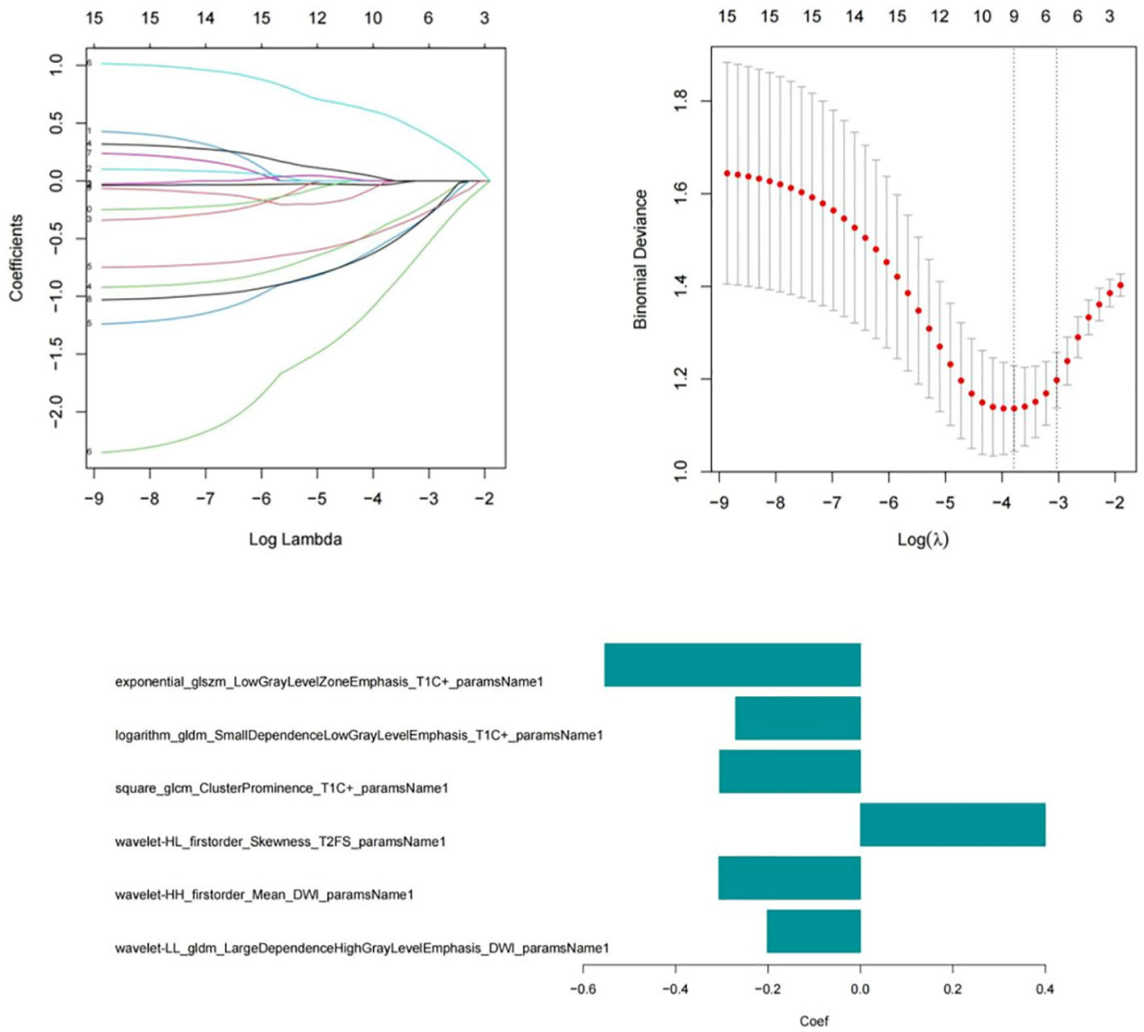
Six radiomic features were screened via the LASSO regression model.

A total of 3372 radiomic features were extracted from all patients’ multimodal MR images, including 1125 features extracted from T2WI-STIR images, 1122 features extracted from CE-T1WI images, and 1125 features extracted from DWI images. First, the features extracted from the MR images were selected via single factor feature selection and correlation analysis, and 15 features were selected. LASSO regression was subsequently used to select the 6 features that were most valuable for predicting the efficacy of chemoradiotherapy against NPC (**Figure 3**), including 1 feature on T2WI-STIR, 3 features on CE-T1WI, and 2 features on DWI.

The formula for calculating the radiomics score is as follows: Score=-0.632-0.667*exponential_glszm_LowGrayLevelZoneEmphasis_T1C-0.893*logarithm_gldm_SmallDependenceLowGrayLevelEmphasis_T1C+0.903*square_glcm_ClusterProminence_T1C-1.007*wavelet-HL_firstorder_Skewness_T2FS-0.659*wavelet-HH_firstorder_Mean_DWI-1.734*wavelet-LL_gldm_LargeDependenceHighGrayLevelEmphasis_DWI. After univariate and multivariate logistic regression analyses of the clinical risk factors, 2 types of clinical information (NLR and T stage, **Table 3**) were ultimately selected. Finally, a clinical model, radiomics model and clinical–radiomics model were established by combining the obtained clinical information and radiomic features.

**Table 3:** Univariate and multivariate logistic regression analyses of clinical information.

Parameter	Univariate Logistic Regression			Multivariate Logistic Regression		
	OR	95% CI	P value	OR	95% CI	P value
NLR	1.57	1.02-2.41	0.04	1.59	1.04-2.52	0.038
PLR	1.00	1.00-1.01	0.217	–	–	–
Age	1.04	1.00-1.09	0.074	–	–	–
Tumor length	0.66	0.29-1.49	0.321	–	–	–
Sex	1.44	0.51-4.08	0.496	–	–	–
Bloody nasal discharge	0.70	0.24-1.98	0.496	–	–	–
LN metastasis	0.50	0.08-3.23	0.47	–	–	–
T stage	2.83	1.07-7.49	0.037	2.96	1.09-8.42	0.036
Clinical stage	1.59	0.57-4.46	0.374	–	–	–

### Model comparison and verification

**Figure 4:**
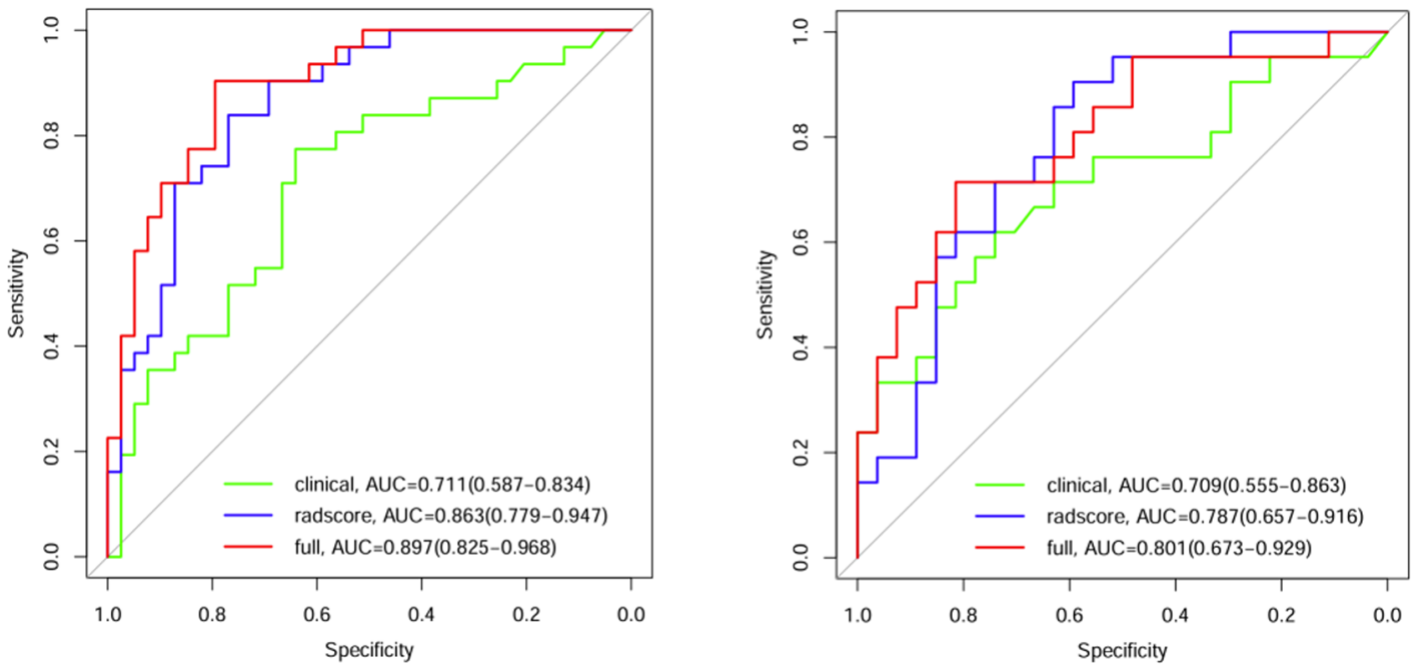
ROC curves for the 3 models in the training group and validation group.

Comparisons were made among the clinical model, the radiomics model and the combined clinical–radiomics model. The AUCs of the training group were 0.711, 0.863 and 0.897, respectively. The 95% CIs were 0.587–0.834, 0.779–0.947 and 0.825–0.968, respectively. The specificities were 0.641, 0.769 and 0.795, respectively. The sensitivities were 0.774, 0.839 and 0.903, respectively. The Youden indices were 0.415, 0.608 and 0.698, respectively. In the validation group, the AUCs were 0.709, 0.787 and 0.801, respectively. The 95% CIs were 0.5555–0.863, 0.6577–0.916 and 0.673–0.929, respectively. The specificities were 0.741, 0.593 and 0.815, respectively. The sensitivities were 0.619, 0.905 and 0.714, respectively. The Youden indices were 0.360, 0.498 and 0.529, respectively (**Table 4**; **Figure 4**).

**Table 4:** Comparison of the groups with the three models.

Groups	AUC (95% CI)	Specificity	Sensitivity	Youden index
Training group				
Clinical model	0.711 (0.587-0.834)	0.641	0.774	0.415
Radiomics model	0.863 (0.779-0.947)	0.769	0.839	0.608
Combined model	0.897 (0.825-0.968)	0.795	0.903	0.698
Validation group				
Clinical model	0.709 (0.555-0.863)	0.741	0.619	0.360
Radiomics model	0.787 (0.657-0.916)	0.593	0.905	0.498
Combined model	0.801 (0.673-0.929)	0.815	0.714	0.529

**Figure 5:**
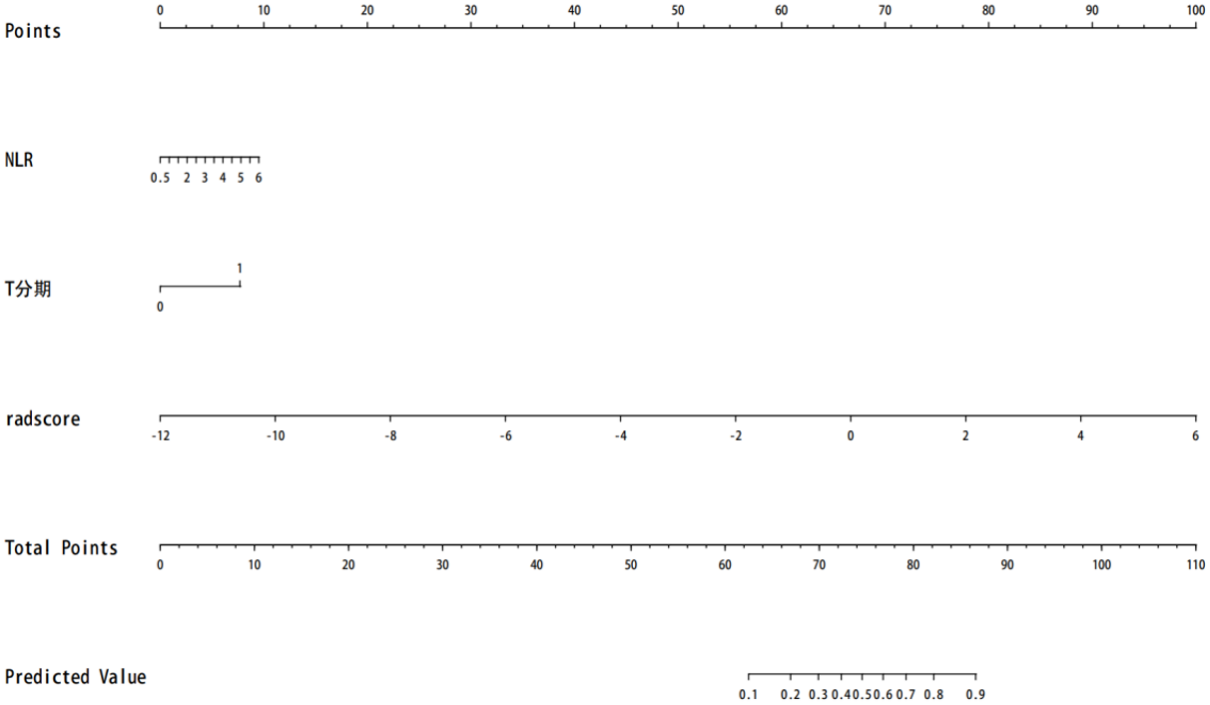
The prognosis of nasopharyngeal carcinoma patients treated with chemoradiotherapy is predicted. Note: T stage—1 represents T3–T4, and 0 represents T1–T2.

**Figure 6:**
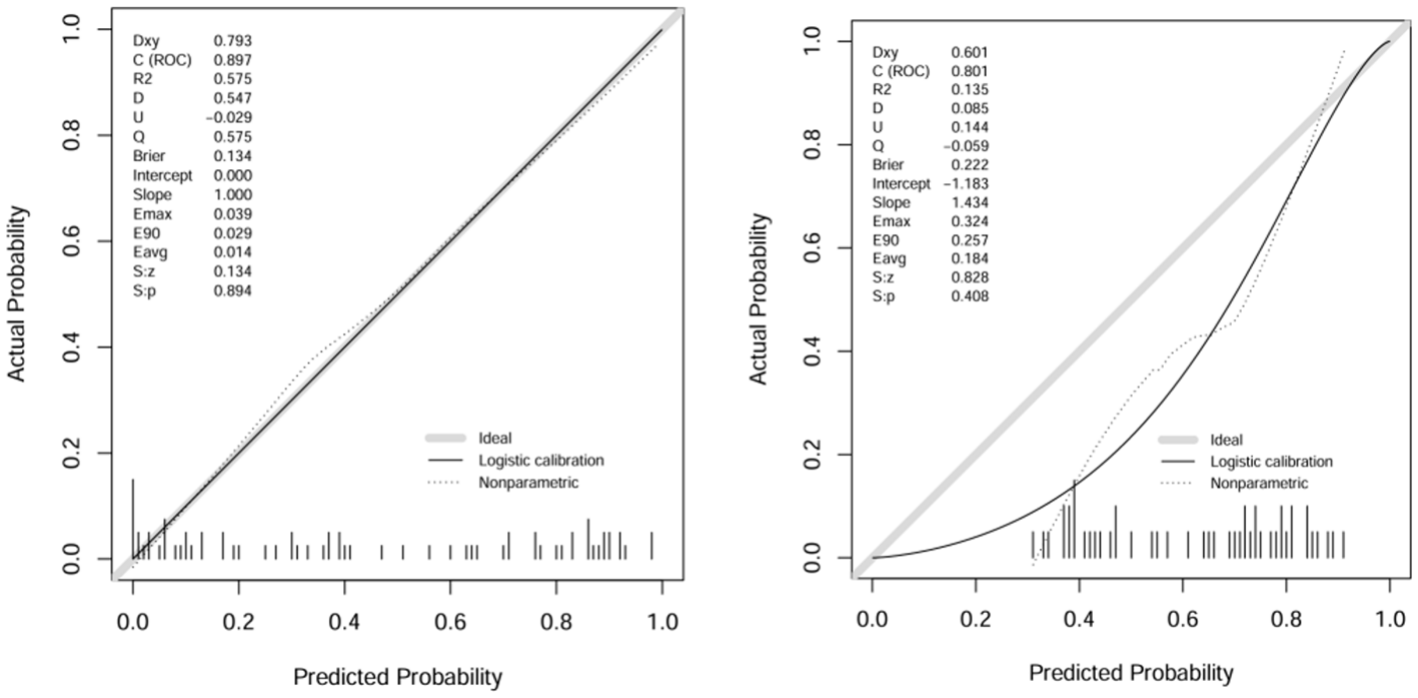
Calibration curves of the training group and validation group with columns.

**Figure 7:**
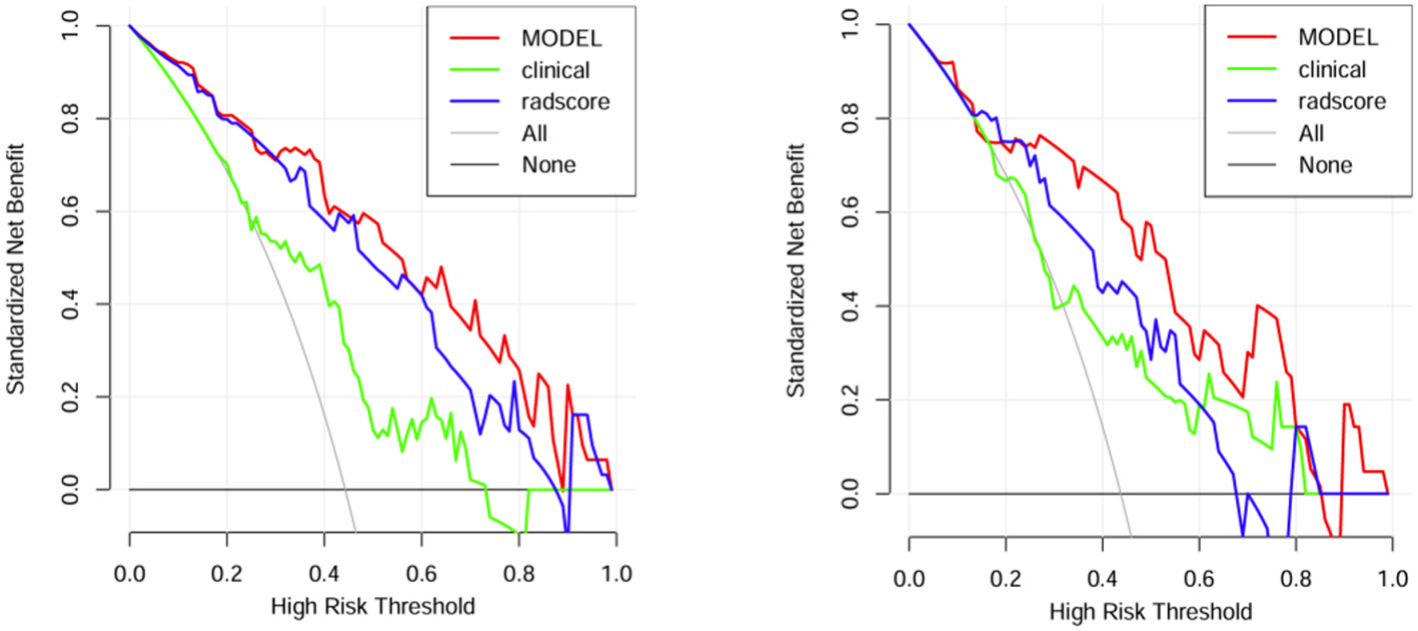
DCA curves of the training group and verification group.

A comparison of the AUC, specificity and sensitivity of the three models constructed above revealed that the predictive efficacy of the clinical–radiomics model for the training group (AUC=0.897) and validation group (AUC=0.801) was greater than that of the single clinical or single radiomics model. Compared with those of the Delong test, the AUCs of the three models were significantly different in both the training group and the validation group (P < 0.05). Finally, a nomogram based on the clinical–radiomics model was constructed to visualize the prediction model (**Figure 5**). The higher the value calculated by this model is, the greater the likelihood that the patient will progress after chemoradiotherapy. In the training group and the validation group, the calibration curve of the nomogram showed good agreement between the probability of predicting the outcome of NPC patients after chemoradiotherapy and the true probability (**Figure 6**). The DCA curve also revealed that, compared with the other two groups of models, the combined model had the greatest net benefit and greater clinical application value (**Figure 7**).

### Kaplan−Meier survival curve analysis

**Figure 8:**
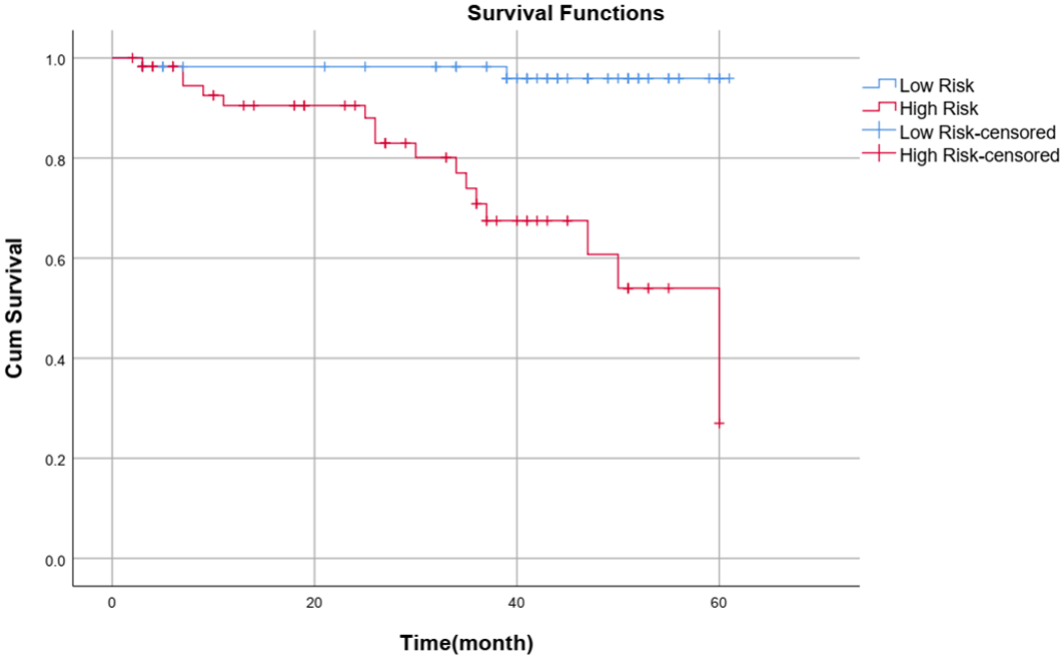
Survival curve of the combined model.

Patients were divided into high-risk and low-risk groups according to the median risk score (-0.46) calculated with the clinical–radiomics model as the cutoff value. Patients with a risk score >-0.46 were classified into the high-risk group, and patients with a risk score ≤-0.46 were classified into the low-risk group. The Kaplan–Meier survival curve analysis revealed that patients in the low-risk group (blue curve) had a better outcome than those in the high-risk group did (red curve) (P<0.001). The survival curve of the combined model is shown in **Figure 8**.

## Discussion

In this study, we validated the possibility of predicting the outcome of chemoradiotherapy in patients with advanced NPC based on a multisequence MRI clinical-radiomics model nomogram. A combined clinical–radiomics model was more accurate in predicting PFS than a single clinical model or single radiomics model. The multidimensional nomogram constructed by combining the rad score, T stage and NLR had a good prediction effect, enabled visualization of the prediction model, and helped to accurately, independently and intuitively predict the outcome of advanced NPC patients. Notably, the median risk score calculated by the combined model was a critical value, and patients were divided into high-risk and low-risk groups. The Kaplan−Meier survival curve revealed that the PFS of the low-risk group was significantly better than that of the high-risk group (P< 0.001).

Owing to the hidden anatomical location of NPC, surgical treatment is generally not used for the initial treatment of NPC. At present, CCRT combined with IC or AC has become the standard treatment for advanced NPC. With advancements in radiotherapy technology and drugs for medical treatment, the clinical efficacy of treatment for NPC patients has gradually improved, but some patients still experience local recurrence or metastasis after initial treatment (19, 20). Predicting the response of patients to chemoradiotherapy before treatment, adjusting the treatment plan in time, achieving standardized and time-effective treatment, reducing the adverse reactions of patients with NPC and improving their quality of life are particularly important. The value of radiomics in determining the prognosis of NPC patients has been proven in many studies (18, 21–23). Sararas et al. (21) retrospectively analyzed 183 patients with NPC. As in this study, each patient was followed up for at least 3 years, and efficient radiomics features were obtained through screening. These features were combined with clinical variables and the TNM stage to establish a model to evaluate patient prognosis. The results showed that multimodal radiomics combined with clinical data had the highest performance. Most of the above NPC radiomics studies used traditional T2WI and CE-T1WI sequences for radiomic features analysis but did not include important DWI sequences, and the image information was incomplete. Tumor image information from multiple sequences can better reflect tumor heterogeneity and radiomic multiparameters. DWI can provide more subvoxel image information about tumor heterogeneity, reflecting the limited Brownian motion and microstructure in the tumor (24, 25). As a routine sequence of MRI scans for NPC, DWI has important reference significance for evaluating the efficacy and prognosis of NPC (26). In this study, we included DWI sequences and extracted important features from them to construct the radiomic models.

By analyzing the combined images of the T2WI-STIR sequence, CE-T1WI sequence and DWI sequence, six radiomic features were obtained after feature extraction, selection and dimensionality reduction, including 1 feature on T2WI-STIR, 3 features on CE-T1WI, and 2 features on DWI. Preliminary results showed that the DWI parameters were equally important. Through the exponential transform, logarithmic transform and square root transform, three radiomic features, all of which are high-order features, were obtained. Three radiomic features were derived via wavelet transform. The wavelet transform is a new transformation analysis method in image processing that has gradually refined signal processing on multiple scales through telescopic translation, thus focusing on the details of the signal. Among the 6 radiomic features extracted in this study, the wavelet transform accounted for the greatest proportion, which may be related to the high soft tissue resolution and obvious detail display of the MR images.

We added economic and convenient indicators such as hematology parameters to improve the convenience and feasibility of the model in clinical practice. Univariate and multivariate logistic regression analyses revealed that the T stage and NLR were independent predictors (P < 0.05). At present, the TNM staging system widely used in the staging of NPC mainly focuses on the length and diameter of the tumor, the depth and breadth of invasion, the location and size of the involved lymph nodes, and the presence of distant metastasis (27). Zhao et al. (28) retrospectively analyzed the clinical characteristics of 527 NPC patients after IMRT. Cox risk regression revealed that the T stage was an important risk factor for local relapse-free survival (LRFS). The application of T stage helps NPC patients achieve higher local tumor control rates and 5-year overall survival (OS) rates during treatment. In tumors and other pathological conditions, the NLR represents the dynamic relationship between innate (neutrophilic) and adaptive (lymphocyte) cellular immune responses (10, 29, 30). Numerous cytokines and angiogenic factors are released by neutrophils during tumor cell growth, angiogenesis, and metastasis (31). Lymphocyte infiltration plays an important role in improving patient prognosis and therapeutic response (32). As a result, the NLR may provide insights into the relationships among tumors, inflammatory responses, and the immune system as a whole. Through a literature review, numerous studies (33–35) have shown that the peripheral blood NLR is an independent risk factor for predicting the outcome of patients with NPC, but most of those studies included only clinical indicators and did not include radiomic characteristics in the analysis. Therefore, we extracted easily available hematological indicators from NPC patients, identified stable and efficient clinical markers, and incorporated them into the radiomics nomogram. Compared with those in other studies, the parameters included in our model are more universal, so the model is more applicable. By measuring relevant parameters before chemoradiotherapy, our nomogram helps to accurately and intuitively predict the outcome of patients with advanced NPC. If NPC patients with a long PFS can be identified before chemoradiotherapy, clinicians can improve the treatment plan and choose a treatment with fewer side effects under the premise of the same overall treatment outcome to improve the quality of life of patients. Conversely, for patients with a short PFS predicted by the model, intensive therapy and combined targeted therapy can be used as appropriate, and poor prognostic factors can be considered.

However, this research still has some shortcomings. First, this study included only patients with stage II–IV NPC, and future studies should consider patients with other disease stages. Second, this study did not distinguish in detail the different effects of IC and AC on the prognosis of patients, and detailed subgroup studies on different chemotherapy drugs should be conducted in the future. Third, the minimum and maximum PFS times of our study were 3 years and 5 years, respectively. In the future, we will include more patients from medical institutions in the same region and further classify the 3-year PFS, 5-year PFS and long-term overall survival of patients. Fourth, with advancements in medical technology, the survival time of patients with NPC has gradually increased, resulting in a limited dataset for the death group in this study. Finally, this study only sketched the two-dimensional ROI at the largest level, and all of them were segmented manually, resulting in the subjectivity of segmented images. At present, semi-automatic and automatic segmentation techniques have been applied in the research of NPC images (36), and three-dimensional and automatic segmentation will be tried in the subsequent research. To this end, we expect to expand the follow-up work and add more data from studies over time to increase the robustness and reliability of the model.

In conclusion, in this study, we combined T stage, NLR, and radiomic features to develop and validate a clinical–radiomics nomogram based on multisequence MRI and clinical risk factors that can effectively predict the outcome of patients with stage II-IV NPC. This model can be used as a noninvasive and beneficial tool to promote the individualized treatment and optimal management of NPC patients, provide new ideas for the establishment of a more comprehensive prognostic model, and provide more auxiliary references for clinical practice.

## Data Availability

The original contributions presented in the study are included in the article/supplementary material. Further inquiries can be directed to the corresponding author.
